# Shared pivotal genome of European and South American polyploid fescues (*Festuca* sp.) uncovered by comparative cytogenetics

**DOI:** 10.1093/aob/mcag261

**Published:** 2026-08-17

**Authors:** Yasmim Dutra Santos, Marco Túlio Mendes Ferreira, David Kopecky, Vânia Helena Techio

**Affiliations:** Department of Biology, Federal University of Lavras (UFLA), 37200-000 Lavras, Minas Gerais, Brazil; Centre of Plant Structural and Functional Genomics, Institute of Experimental Botany of the Czech Academy of Sciences, 77900 Olomouc, Czech Republic; Department of Biology, Federal University of Lavras (UFLA), 37200-000 Lavras, Minas Gerais, Brazil; Centre of Plant Structural and Functional Genomics, Institute of Experimental Botany of the Czech Academy of Sciences, 77900 Olomouc, Czech Republic; Department of Biology, Federal University of Lavras (UFLA), 37200-000 Lavras, Minas Gerais, Brazil

**Keywords:** Genomic *in situ* hybridization, wild grasses, pivotal genome, comparative cytogenetics, genome size, *Festuca ulochaeta* Steud, *Festuca fimbriata* Ness, *Festuca arundinacea* Schreb

## Abstract

**Background and Aims:**

Polyploidization is one of the major evolutionary forces in grasses (Poaceae), yet the genomic relationships between polyploid species across different continents remain poorly understood. Here, we aimed to determine whether phylogenetically distinct South American allohexaploid fescues, *Festuca ulochaeta* Steud. (subg. *Subulatae*) and *F. fimbriata* Ness. (subg. *Mallopetalon*), retain a shared ancestral (pivotal) genome with the broad-leaved Eurasian fescues of subg. *Schedonorus*.

**Methods:**

We used comparative cytogenetics (genomic *in situ* hybridization) coupled with flow cytometry.

**Key Results:**

Flow cytometry confirmed that both species are hexaploid (2*n* = 6*x* = 42) with genome sizes of 10.84 and 11.59 pg/2C, respectively. *In situ* hybridization demonstrated that *F. ulochaeta* and *F. fimbriata* are closely related to each other, and that the genome of *F. ulochaeta* is closely related to one of the diploid genomes of the tetraploid *F. glaucescens*, the tetraploid progenitor of hexaploid tall fescue (*F. arundinacea*). The *F. ulochaeta* DNA probe consistently labelled 14 chromosomes in tetraploids *F. mairei*, *F. apennina* and *F. glaucescens*, in both (Continental and Mediterranean) morphotypes of hexaploid *F. arundinacea* and in octoploid *F. atlantigena*, indicating a shared pivotal diploid genome linking South American and Eurasian lineages. This trans-continental genomic affinity supports the role of South America as a secondary centre of *Festuca* diversification and implies that an ancestral diploid lineage colonized the continent prior to, or concurrent with, allopolyploidization.

**Conclusions:**

We revealed, for the first time, the genomic connection between polyploid South American and European fescues. This has direct implications for understanding the evolution of the haplo-insufficient meiotic pairing regulator present in polyploid fescues and for exploiting South American germplasm in grass breeding.

## INTRODUCTION

Polyploidy is one of the main mechanisms of plant evolution (Wendel, 2015; Van de Peer *et al*., 2021). While all angiosperms underwent at least one round of whole genome duplication (WGD) during their evolutionary history, a majority returned to the diploid level via a process called diploidization (Mandáková and Lysak, 2018). Still, about 15–24 % of Angiosperms are polyploids, with large differences across families (Wood *et al*., 2009; Barker *et al*., 2016). Among them, the family Poaceae, which includes many crops and economically important species, is generally polyploidy-prone (Cheng *et al*., 2022). However, there is wide variability in the frequency of polyploids among different genera of this family. While some genera (e.g. *Lolium*) lack any polyploid species or cytotypes, they are highly abundant in others (e.g *Festuca* and *Triticum*) (Cheng *et al*., 2016; Humphreys and Zwierzykowski, 2020). Why polyploids prevail in some genera and are absent in others is a matter of debate and hypotheses (Wendel, 2015; Nieto Feliner *et al*., 2020; Barker *et al*., 2024). To establish as a long-lasting population, polyploids have to solve several issues connected with the doubling of their nuclear genome, among which the presence of more than two chromosomes able to pair and recombine during meiosis is probably the most important (Twyford *et al*., 2025; Shi and van de Peer, 2026).

The genus *Festuca* Tourn. ex L. (Poaceae) is one of the largest among Poaceae, comprising seven subgenera with over 650 species (POWO, 2022) distributed on all continents, except for Antarctica (Watson and Dallwitz, 1992). It originated in the early Miocene ca. 20 million years ago in the region around the Mediterranean Sea with South America as a secondary centre of *Festuca* diversification (Dubcovsky and Martinez, 1992; Darbyshire *et al*., 2003; Stančík, 2003; Fernández Pepi *et al*., 2012; Ospina and Picca, 2019). The entire genus is described as paraphyletic, which corresponds to its ancestral evolutionary position within the subtribe Loliinae (Catalán *et al*., 2004). There are two main evolutionary lineages: fine-leaved fescues (with two main lineages representing complexes *F. rubra* and *F. ovina*) and broad-leaved fescues tightly linked to other genera (*Lolium*, *Vulpia* and *Micropyropsis*) (Inda *et al*., 2014). Some fescue species (such as *F. fimbriata*) display incongruence between morphological characteristics (broad leaves) and phylogenetic positioning (within fine-leaved fescues) (Moreno-Aguilar *et al*., 2022).

Besides diploid species (2*n* = 2*x* = 14), the genus displays higher ploidies up to dodecaploidy (2*n* = 12*x* = 84; Šmarda and Stančík, 2006; Loureiro *et al*., 2007; Šmarda *et al*., 2008; The Chromosome Counts Database – accessible at https://ccdb.tau.ac.il/). Diploids occur mainly in Europe and west-central Asia, with some diploids listed in North America (with close relationship to East Asian species, from where they probably migrated through the Bering Strait; Šmarda and Stančík, 2006), but there are no reports of them in the southern hemisphere (Inda *et al*., 2008). The world-wide existence of a large number of polyploid lines raises the question of whether at least some of them share part of their evolutionary history and contain one or more ancient ancestral diploid genomes. Such a pivotal genome would thus be detectable in present-day polyploid species and would also indicate the potential for intercrossing among these species, a significant aspect for the breeding of new crop cultivars. Polyploids in fescues originated by both auto- and allopolyploidization (Loureiro *et al*., 2007; Šmarda *et al*., 2008). The most detailed knowledge of genome composition and evolution in polyploid fescues comes from studies of cultivated and economically important taxa including tall fescue (*F. arundinacea* Schreb.) from subg. *Schedonorus*. This species is hexaploid (2*n* = 6*x* = 42), having originated by hybridization of *F. glaucescens* Boiss. (4*x*) and *F. pratensis* Huds. (2*x*). Thus, this species, used often for forage and turf for its good persistence and tolerance to abiotic stress conditions (Jauhar, 1993; Rognli *et al*., 2010; Harper *et al*., 2018; Černoch and Kopecký, 2020) is of allopolyploid origin, composed of three diploid genomes (FgFg′Fp). It carries a diploidizing chromosome pairing regulator, inherited from the parental *F. glaucescens*, a species endemic to the mountainous regions of the Pyrenees and Alps (Jauhar, 1975; Kopecký *et al*., 2009). The origin of *F. glaucescens* is so far unknown. While its two diploid genomes cannot be discriminated by genomic *in situ* hybridization (GISH), it is probably of allopolyploid origin (Kopecký *et al*., 2009). The genome location of the pairing regulator of *F. glaucescens* is also unknown. However, identification and further characterization of this regulator would allow its utilization in practice. Interspecific hybridization is often used in grass breeding, but its usefulness is frequently hampered by genomic instability of the hybrids (Kopecký and Studer, 2014). It was found that newly developed interspecific fescue hybrids or intergeneric hybrids of fescues with closely related ryegrasses (*Lolium* L.) display homoeologous chromosome pairing and recombination. This leads to unbalanced segregation of parental chromosomes during anaphase I and, in turn, highly variable and genomically unstable progeny (Zwierzykowski *et al*., 1998; Kopecký *et al*., 2006).

Polyploid species from subg. *Schedonorus*, all native to Eurasia and northern Africa, share phylogenetic histories with species beyond that region. Namaganda and Lye (2008) found that the central African species *F. simensis* is closely related to polyploid fescues from subg. *Schedonorus*. In the present study, we aimed to test if such phylogenetic relationships exist between Eurasian and South American polyploid fescues and if they potentially retain a shared ancestral (pivotal) genome. Because there are no diploids in South America, we assume that polyploids migrated there from Europe and we aim to trace the pivotal genomes shared by both South American and European diploid and polyploid lineages of the genus *Festuca*. To fill these gaps in our knowledge, we used comparative molecular cytogenetics with South American *F. ulochaeta* Steud. and *F. fimbriata* Ness. and European representatives of subg. *Schedonorus*. These two South American species were deliberately selected, because they combine three characteristics: (1) they represent distinct evolutionary lineages (subg. *Subulatae* as a representative of broad-leaved fescues and *Mallopetalon* as a representative of fine-leaved fescues based on phylogenetic studies; Moreno-Aguilar *et al*., 2022) within the South American radiation of *Festuca*; (2) both are allopolyploid (2*n* = 6*x* = 42), similar as some of the Eurasian broad-leaved polyploid fescues; and (3) they are among the few South American species with well-characterized ecotypes. These characteristics make them excellent models to study phylogenetic relationships among species from South America and Europe. Similar to *F. arundinacea*, both species prefer wet habitats; *F. ulochaeta* grows in the humid forests of S and SE Brazil and NE Argentina between 700 and 2200 m. In Colombia, Peru and Venezuela this species occurs in forest clearings, streams margins and roadsides in Andean forests (Cordillera Oriental and Aragua, Merida) between 2600 and 3100 m a.s.l. *F. fimbriata* is restricted to the area between northern Uruguay, northeastern Argentina, and south and southeastern Brazil, in forest openings, wet depressions and swamps from sea level up to 2400 m a.s.l. (Stančík and Peterson, 2007; POWO, 2022). Rather than aiming for exhaustive taxonomic sampling, our strategy was designed to maximize the ability to detect genomic signatures shared across independent South American lineages and to evaluate their relationship with the well-characterized Eurasian polyploid complex.

## MATERIAL AND METHODS

### Plant material

The analyses involved native South American and Eurasian species. Tillers of *Festuca ulochaeta* and *F. fimbriata* were collected in the Aparados da Serra National Park, Itaimbezinho, municipality of Praia Grande, state of Santa Catarina (SC), Brazil (29°09′33.0″S, 50°04′45.6″W) and in the municipality of Bom Jesus, state of Rio Grande do Sul (RS), Brazil (28°36′02.3″S, 50°24′29.7″W), respectively. Among the species of Eurasian origin, *Festuca pratensis* was obtained from the USDA germplasm bank and *Festuca arundinacea* from the company PGW Seeds, municipality of Porto Alegre, RS; all others are in possession of the Institute of Experimental Botany CAS, Czech Republic (for details on plant material, see Supplementary Data Table S1A).

### Chromosome preparation and *in situ* probing

Tips of actively growing roots were collected and pre-treated with cyclohexamide (88.9 µm) and amiprophos-methyl (APM) (4 µm) (2:1), for 2:30 h at room temperature, fixed in the methanol/acetic acid mixture (3:1) and stored in 90 % ethanol. Cell wall digestion was performed in an enzyme mix containing ‘Onozuka R10’ cellulase (0.7 %), Sigma-Adrich cellulase (0.7 %), Sigma-Aldrich pectolyase (1 %) and Sigma-Aldrich cytohelicase (1 %) for 1:30 h at 37 °C. Slides were prepared using the cell dissociation technique (De Carvalho and Saraiva, 1993) and dried at room temperature.

Genomic DNAs (gDNAs) were obtained using the CTAB protocol (Doyle and Doyle, 1990). For GISH, probes were made from gDNA with a concentration of 40 ng µL^–1^ labelled with digoxigenin-12-dUTP or biotin-16-dUTP by nick translation at 15 °C for 1:30 h. The 18S rDNA probe was used for identification of the 35S rDNA region and it was obtained by PCR using primers NS1 (5′-GTAGTCATATGCTTGTCTC-3′) and NS4 (5′-CTTCCGTCAATTCCTTTAAG3′) amplified from the genome of *Oryza sativa* L. (White *et al*., 1990), and subsequently labelled by nick translation with digoxigenin-12-dUTP or biotin-16-dUTP.

For GISH, slides were initially post-fixed with 4 % formaldehyde for 10 min, washed in 2× SSC for 5 min and dehydrated in an ethanol series (70, 90 and 100 %) for 5 min each. The hybridization mix (20 µL) was prepared according to Schwarzacher and Heslop-Harrison (2000), with modifications: 10 µL 100 % formamide, 2 µL 20× SSC pH 7.0, 2 µL labelled probe, 4 µL dextran sulphate (50 %) and 2 µL ultrapure water. The ratio of probe to blocking DNA was 1:150 (Kopecký *et al*., 2008). The mixture was applied to chromosome preparations, and both were denatured at 80 °C for 3 min (Masoudi-Nejad *et al*., 2002). The hybridization process was in a humid chamber at 37 °C for 48 h. After washing with 2× SSC, probes were detected using anti-digoxigenin-rhodamine or streptavidin-Alexa Fluor 488 at 37 °C for 1 h. Chromosomes were counterstained with 2 μg L^–1^ DAPI in Vectashield.

Images were captured using a QImaging Retiga EXi CCD camera attached to an Olympus BX60 epifluorescence microscope, using filters for DAPI (330–385 nm/420 nm), rhodamine (400–410 nm/455 nm) and Alexa Fluor 488 (490 nm/525 nm). The best images were processed for brightness and contrast adjustments in Adobe Photoshop® 23.5.2. Chromosome pairs of *F. fimbriata* were identified based on morphometric data together with the patterns obtained with self-GISH and *F. ulochaeta* gDNA as blocking DNA, and the karyotype described by Ferreira *et al*. (2020). Chromosomal measurements were performed using the free software Karyotype (Yu *et al*., 2015; Altinordu *et al*., 2016) in five metaphases (Supplementary Data Table S1B). The parameters used were chromosome total length (CTL; µm), long arm length (L; µm), short arm length (S; µm), arm ratio (AR; L/S), centromeric index (CI; S × 100/CTL) and total length of the haploid set (TLHS; µm). The karyotype was assembled using Adobe Photoshop® 23.5.2.

#### Genome size estimation

We estimated the genome size of *F. ulochaeta* and *F. fimbriata* by flow cytometry following Doležel *et al*. (2007). Nuclear suspensions were prepared by simultaneous chopping of 50 mg leaf tissues of *Pisum sativum* L. ‘Ctirad’, which was used as an internal reference standard with 2C = 9.09 pg (Dolezel, 1997). The tissues were homogenized with a razor blade in a Petri dish containing 0.5 mL of Otto I solution (0.1 m citric acid, 0.5 % Tween 20; Otto, 1990). The nuclear suspension was filtered through 42-µm nylon mesh and stained with 1 mL Otto II solution (0.4 m Na_2_HPO_4_.12H_2_O) containing 2 mg mL^–1^ β-mercaptoethanol, 50 µL mL^–1^ propidium iodide and 50 µL mL^–1^ RNase IIA. Samples were stained for 10 min at room temperature and analysed using a FACSCalibur cytometer (Becton Dickinson). At least 5000 events were analysed per sample and only histograms with a coefficient of variation of G0/G1 peaks lower than 3.0 % were accepted. The analysis was repeated on three different days.

## RESULTS

### Genome sizes and patterns of comparative GISH in South American *Festuca* species

Both *F. fimbriata* and *F. ulochaeta* had 2*n* = 6*x* = 42 chromosomes with genome sizes of 11.59 pg/2C (CV = 1.73 %) and 10.84 pg/2C (CV = 1.87 %), respectively (Table 1). GISH revealed close phylogenetic relationships of the chromosomes from both species. The *F. ulochaeta* probe labelled all *F. fimbriata* chromosomes, except the (peri)centromeric and (sub)telomeric regions and four sites of 18S rDNA (Fig. 1A, B). The pattern of *F. fimbriata* gDNA on *F. ulochaeta* chromosomes was similar, with the (peri)centromeric and two sites of 18S rDNA unlabelled (Fig. 1C). Low hybridization at the (peri)centromeric regions need not indicate lower genome collinearity, but may reflect reduced accessibility of the probe to the presumably more condensed chromatin. A test in *F. fimbriata* with self-GISH (hybridization of *F. fimbriata* total genomic DNA to *F. fimbriata* chromosomes) produced homogeneous signal spread over all 42 chromosomes except the (peri)centromeric regions and regions of 18S rDNA (Fig. 1D). The close phylogenetic relationship between the two South American fescue species was further supported by the use of unlabelled blocking DNA of *F. ulochaeta*. This *F. ulochaeta* blocking DNA effectively blocked the hybridization of the *F. fimbriata* gDNA probe along all chromosomes except (peri)centromeric, (sub)telomeric and 18S rDNA loci (Fig. 1E). Using this combination (self-probe and blocking DNA from *F. ulochaeta*), it was possible to identify individual chromosomes based on different intensities of the signal, especially in (sub)telomeric regions of *F. fimbriata* chromosomes (Fig. 1E).

**Fig. 1.**
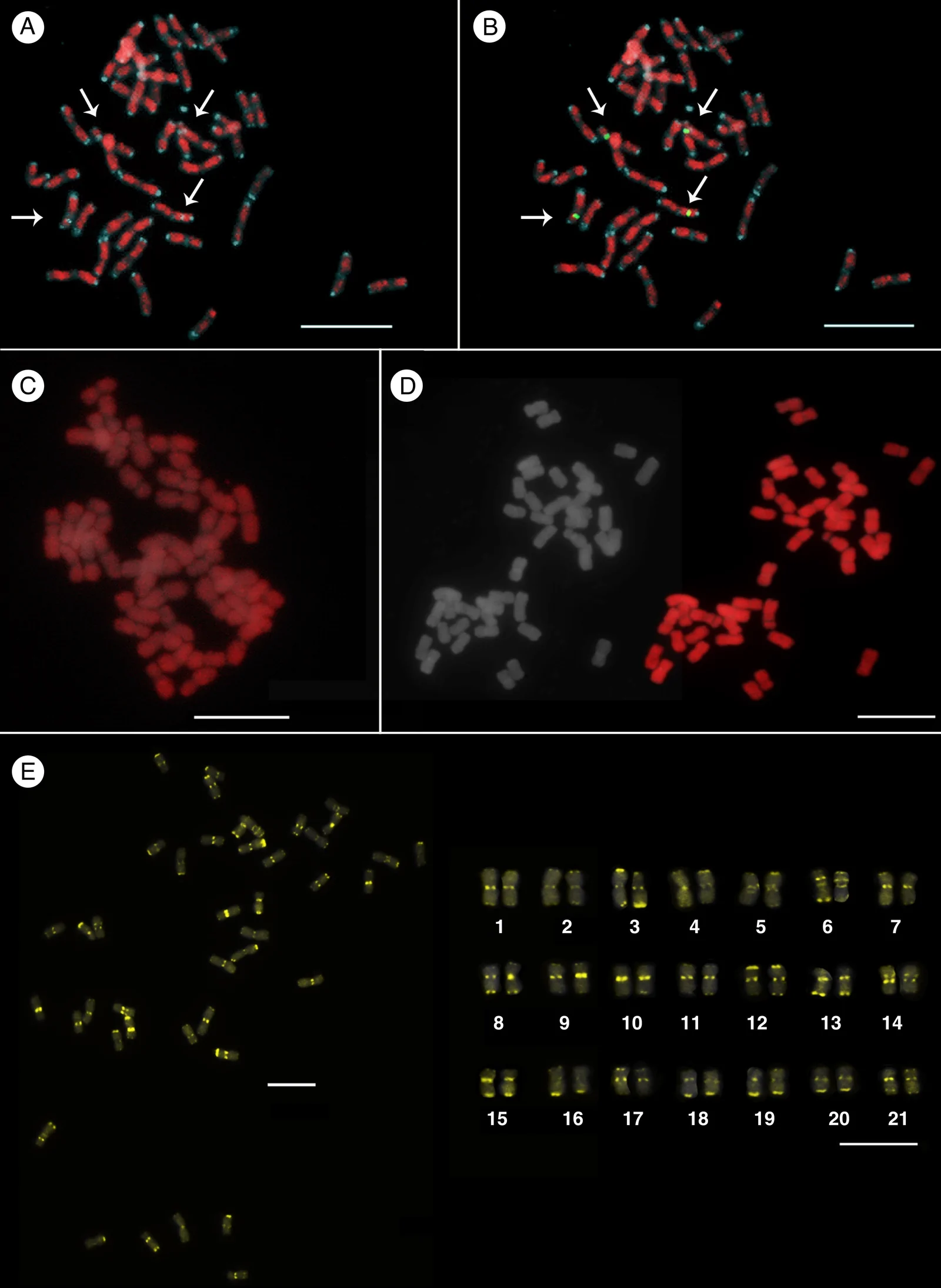
Comparative GISH between South American species *F. fimbriata* and *F. ulochaeta* (2*n* = 6*x* = 42). (A) *In situ* hybridization on *F. fimbriata* chromosomes with *F. ulochaeta* gDNA probe (red) and (B) reprobing of the same chromosome spread with 18S rDNA (yellow; arrows) and *F. ulochaeta* gDNA (red) probes. (C) *In situ* hybridization in *F. ulochaeta* chromosomes with *F. fimbriata* gDNA probe (red). (D) Self-GISH on *F. fimbriata* chromosomes with *F. fimbriata* gDNA probe (red; right part of the subpanel) and the same metaphase spread counterstained with DAPI without displaying signal (left part). (E) Self-GISH on *F. fimbriata* chromosomes with *F. fimbriata* gDNA probe and *F. ulochaeta* blocking DNA (left) with ordered karyogram (right). Chromosomes were counterstained with DAPI (grey pseudocolour). Bars = 10 µm.

**Table 1.** Nuclear genome size of South American *Festuca* species.

Species	2C value (pg), mean ± s.d.	1C value (pg)	1C value (Mb)	1Cx value (pg)
*F. fimbriata* (6*x*)	11.59 ± 0.2	5.79	5663	1.93
*F. ulochaeta* (6*x*)	10.84 ± 0.2	5.42	5301	1.81

1C value: gametic DNA content, 1Cx: monoploid genome size.

### Comparative GISH of South American and Eurasian *Festuca* species

Except for (peri)centromeric and (sub)telomeric regions of some chromosomes, all 42 *F. fimbriata* chromosomes were intensely labelled by the *F. arundinacea* (2*n* = 6*x* = 42) probe (Fig. 2A). In the reciprocal GISH, the *F. fimbriata* probe hybridized with all 42 *F. arundinacea* chromosomes but with weaker or absent signal in the (peri)centromeric regions of some chromosomes (Fig. 2B). As with blocking DNA from *F. ulochaeta*, blocking DNA from *F. arundinacea* effectively eliminated hybridization of the *F. fimbriata* probe to all *F. fimbriata* chromosomes. This combination (*F. fimbriata* probe and *F. arundinacea* blocking DNA) also produced strong bands in (sub)telomeric regions, but generally did not produce hybridization bands in (peri)centromeric and 18S rDNA regions. On the other hand, there were two more bands in interstitial (distal) regions of a pair of *F. fimbriata* chromosomes (Fig. 2C). These distinct bands indicate that both *F. ulochaeta* and *F. arundinacea* blocking DNAs do not hybridize with the (peri)centromeric and (sub)telomeric regions of the *F. fimbriata* chromosomes, whose sequences are known to be the most evolving (Steckenborn and Marques, 2025).

**Fig. 2.**
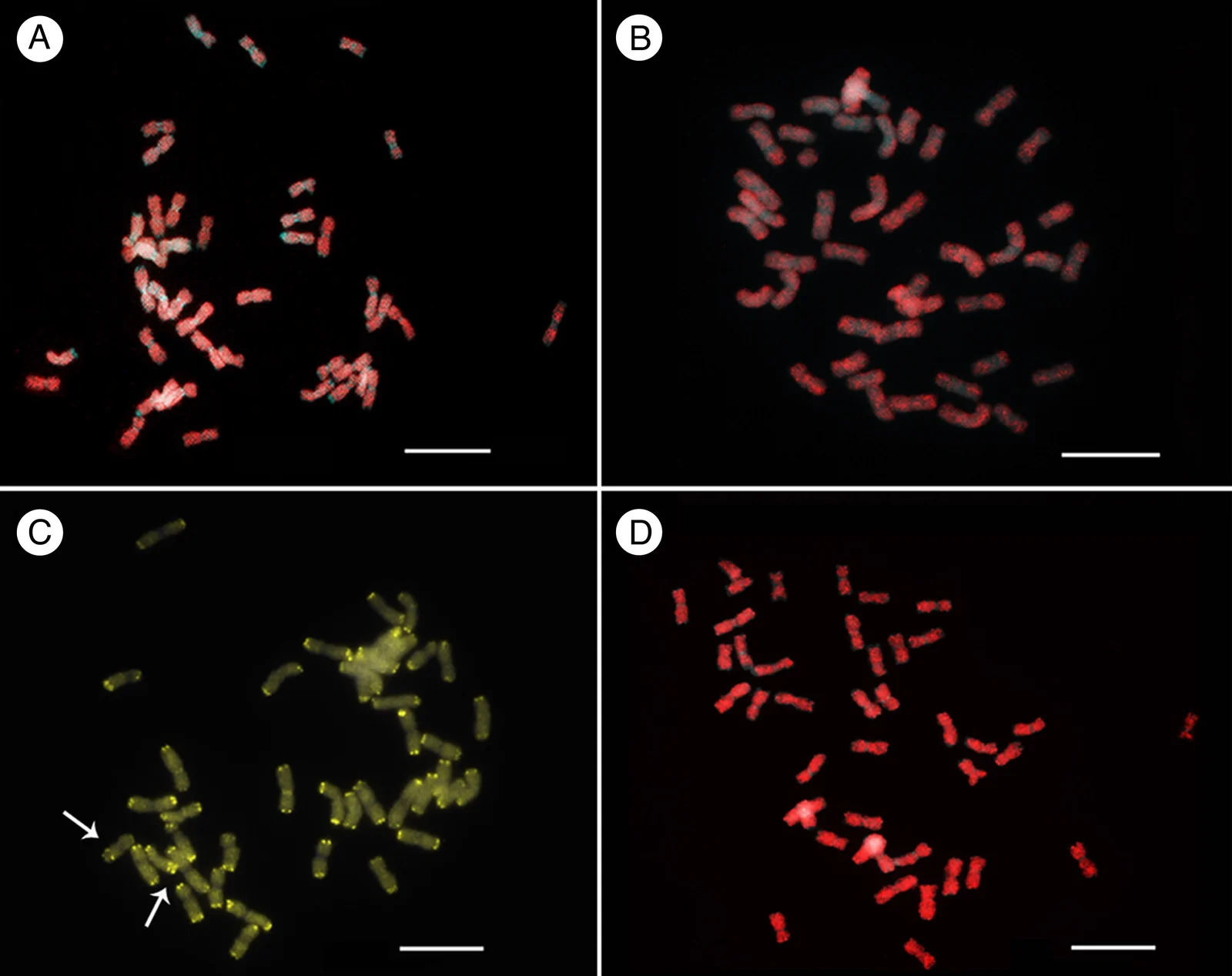
Comparative cytogenetic analysis between *Festuca fimbriata* and Eurasian fescue species from subg. *Schedonorus*. (A) GISH on mitotic metaphase plates of *F. fimbriata* chromosomes with *F. arundinacea* gDNA probe (red); chromosomes were counterstained using DAPI (cyan). (B) Reciprocal experiment on *F. arundinacea* metaphase spread with *F. fimbriata* gDNA probe (red); chromosomes were counterstained using DAPI (cyan). (C) Self-GISH on *F. fimbriata* metaphase chromosome spread with *F. fimbriata* gDNA probe (yellow) and *F. arundinacea* blocking DNA; chromosomes were counterstained using DAPI (grey). Note subtelomeric signals indicated by white arrows. (D) GISH on mitotic metaphase plates of *F. fimbriata* chromosomes with *F. pratensis* gDNA probe (red). Chromosomes were counterstained using DAPI (grey). Bars = 10 µm.

The probe from gDNAs of *F. pratensis* Huds. (2*n* = 2*x* = 14) produced dispersed signal over all *F. fimbriata* chromosomes, again with the exception of (peri)centromeric and (sub)telomeric regions in some chromosomes (Fig. 2D). In tests of the affinity of hexaploid *F. ulochaeta* to one of the diploid genomes of the polyploid fescues from subg. *Schedonorus*, probes from *F. ulochaeta* and *F. pratensis* labelled 14 chromosomes each in tetraploid *F. mairei* (Fig. 3A), *F. apennina* (Fig. 3B), and both the Continental and Mediterranean morphotypes of hexaploid *F. arundinacea*, leaving 14 chromosomes of both morphotypes of *F. arundinacea* unlabelled (Fig. 3D, E). In *F. glaucescens*, the probe from *F. ulochaeta* also labelled 14 chromosomes, but the probe from *F. pratensis* did not produce any signal (Fig. 3C). In *F. atlantigena*, 14 chromosomes were labelled with the *F. ulochaeta* probe, while the remaining 42 chromosomes were labelled with the *F. pratensis* probe (Fig. 3F). This indicates that one diploid genome of polyploid broad-leaved fescues of subg. *Schedonorus* is closely related to the genome of *F. ulochaeta*. We further elucidated whether this close phylogenetic relationship is with each of the three diploid genomes of hexaploid *F. ulochaeta*. Probes made from *F. arundinacea* and *F. glaucescens* produced dispersed signals over all 42 chromosomes of *F. ulochaeta*, with weaker (or absent) signals in (sub)telomeric and (peri)centromeric regions of some chromosomes and the sites of 18S rDNA (Fig. 3G, H).

**Fig. 3.**
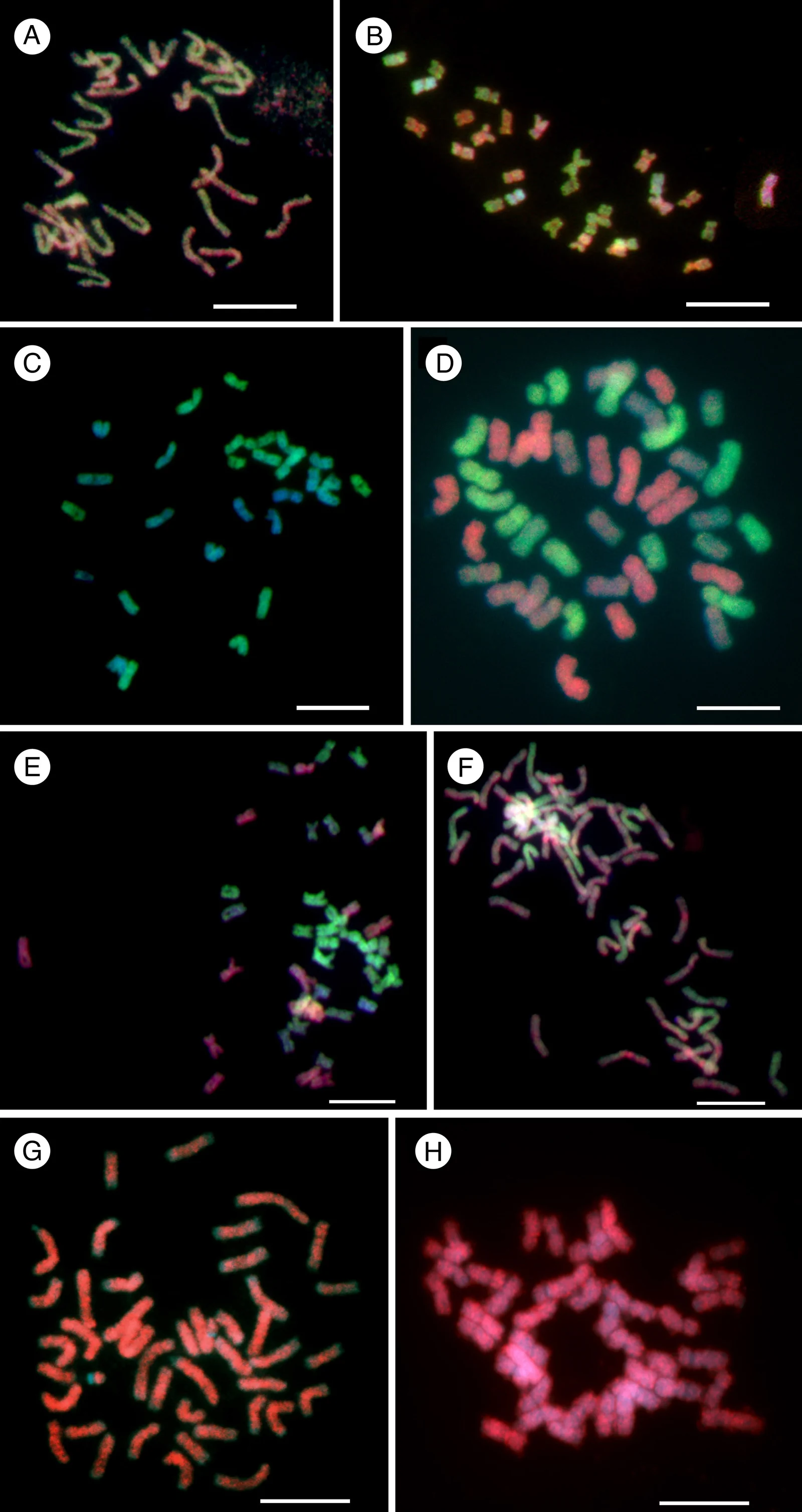
Comparative cytogenetic analyses between *Festuca ulochaeta* and polyploid fescues from subg. *Schedonorus*. *In situ* hybridization on metaphase chromosome spreads of *F. mairei* (A), *F. apennina* (B), *F. glaucescens* (C), *F. arundinacea* (Continental morphotype; D), *F. arundinacea* (Mediterranean morphotype; E) and *F. atlantigena* (F) with *F. ulochaeta* gDNA (green) and *F. pratensis* gDNA (red) probes. *In situ* hybridization on *F. ulochateta* chromosome spreads with the probes made from gDNAs of *F. arundinacea* (G) and *F. glaucescens* (H). Chromosomes were counterstained with DAPI [blue(ish)]. Bars = 10 µm.

Next, we estimated the size of the pivotal genome shared among polyploid species using the genome sizes of the investigated species (Kopecký *et al*., 2010; Zwyrtková *et al*., 2020) and their chromosome numbers. The monoploid size of the pivotal genome ranged from 2.43 pg in *F. mairei* to 2.81 pg in *F. arundinacea*. We further refined these estimates by normalizing them according to the chromosome lengths of this genome in each of these species. Following this normalization, the monoploid size values were somewhat higher: 2.55 pg for *F. mairei*, 2.77 pg for *F. glaucescens* and 2.87 pg for *F. arundinacea* (Supplementary Data Table S1C). This suggests that the pivotal genome is probably slightly larger than the other diploid genomes in polyploids, except in *F. arundinacea*, where the genome derived from *F. pratensis* was the largest.

## DISCUSSION

### Genome sizes in South American species

According to the classification proposed by Leitch *et al*. (2005), genomes of *F. fimbriata* and *F. ulochaeta* are considered medium-sized. However, their monoploid genomes appear to be among the smallest in *Festuca* species (Šmarda *et al*., 2008). The difference in the nuclear DNA content between *F. fimbriata* and *F. ulochaeta* as measured by flow cytometry here (about 0.37 pg, representing a 6.8 % difference) was somewhat smaller than the rough estimation of the genome size by the measurements of chromosome lengths (14.4 % difference; Ferreira *et al*., 2020). This might be caused by differences in chromosome condensation or by larger errors in measuring chromosome lengths. The genome size has never been estimated for *F. fimbriata*; estimates for *F. ulochaeta* vary across studies. Using Fuelgen densitometry, Schifino and Winge (1983) estimated its genome size to 1C = 7.1 pg, while flow cytometry with propidium iodide staining produced lower values of 1C = 5.42 pg in this study and 1C = 4.52 pg in Šmarda and Stančík (2006). These estimates are much smaller than the estimates for allohexaploid *F. arundinacea* [8.41 pg/1C by Rocha *et al*. (2017) and 8.42 pg/1C by Zwyrtková *et al*. (2020)], a species sharing one of its three diploid genomes with *F. ulochaeta*. What causes such differences between these two species is unclear and we can only hypothesize that the bigger genome size could be due to (1) larger genomes of the two non-shared parental diploid genomes in *F. arundinacea*, (2) the proliferation of repetitive elements, presumably retrotransposons such as *Gypsy* and *Copia* long terminal repeats (LTRs), after the origination of *F. arundinacea* and/or (3) the downsizing of the genome of *F. ulochaeta*. Indeed, the genome of *F. pratensis*, one of the parental genomes of *F. arundinacea* but absent from *F. ulochaeta*, is larger than the straight 1/3 of the genome of *F. arundinacea* (Kopecký *et al*., 2009; Zwyrtková *et al*., 2020). Similarly, *Ty3/Gypsy* LTR retroelements are significantly more abundant in the genome of *F. pratensis* compared to that of *F. glaucescens*, and it seems that this family proliferated more in the genome of *F. arundinacea* after the species formation (Zwyrtková *et al*., 2020).

### Phylogenetic relationships among South American and Eurasian polyploid fescues

The genus *Festuca* represents one of the most polyploidy-prone genera in the Poaceae, with ploidy levels ranging from diploid (2*n* = 2*x* = 14) to dodecaploidy (2*n* = 12*x* = 84) (Šmarda and Stančík, 2006; Loureiro *et al*., 2007). In fescues, polyploidization has occurred predominantly via allopolyploidization, resulting in species with composite genomes inherited from two or more distinct progenitor lineages (Loureiro *et al*., 2007; Kopecký *et al*., 2009; Dierking *et al*., 2015). Allopolyploid speciation is particularly prevalent in subg. *Schedonorus*, which includes agronomically important broad-leaved fescues. The well-characterized origin of hexaploid tall fescue (*F. arundinacea* Schreb., 2*n* = 6*x* = 42) from *F. glaucescens* Boiss. (4*x*) and *F. pratensis* Huds. (2*x*) has established a clear framework for understanding the genome composition in polyploid fescues (Humphreys *et al*., 1995). The present study extends this framework by revealing, for the first time, that Eurasian polyploid fescues of subg. *Schedonorus* share one of their diploid genomes with the polyploids from South America.

GISH using *F. ulochaeta* probe on tetraploids *F. mairei* and *F. apennina* consistently labelled 14 out of 28 chromosomes, while the remaining 14 were specifically labelled by the *F. pratensis* probe. Similarly, in both Continental and Mediterranean morphotypes of hexaploid *F. arundinacea*, the *F. ulochaeta* probe labelled 14 chromosomes, complemented by *F. pratensis* signal on another 14, with 14 chromosomes remaining unlabelled. This unlabelled set probably corresponds to a third diploid genome for which the identity of the diploid progenitor has not yet been fully resolved. In *F. glaucescens*, the tetraploid progenitor of *F. arundinacea*, the *F. ulochaeta* probe labelled 14 chromosomes in the absence of any signal from the *F. pratensis* probe. This result indicates that the diploid genome shared between *F. ulochaeta* and *F. glaucescens* was subsequently transmitted to *F. arundinacea* upon hexaploidization. Crucially, the dispersed signal pattern produced by *F. arundinacea* and *F. glaucescens* probes across all 42 *F. ulochaeta* chromosomes suggests that the phylogenetic affinity extends across the entire genome of the South American species rather than being restricted to a single diploid genome, pointing to a shared ancestral diploid genome as the pivotal element linking these geographically distant lineages.

Evolution of the polyploid fescues is a complex interweaving of general processes and singular events that have occurred over long time periods (Torrecilla and Catalán, 2002; Catalán *et al*., 2004; Catalán, 2006). The GISH data collectively point to the existence of a pivotal diploid genome shared by all investigated species (our data; Humphreys *et al*., 1995; Kopecký *et al*., 2009; Dierking *et al*., 2015), a genome that is phylogenetically closely related to all three diploid genomes of the South American hexaploids and one of the diploid genomes in the Eurasian polyploids. The identity of the true diploid progenitor of this pivotal genome remains unknown and its elucidation represents an important goal for future work. Diploid *Festuca* species are restricted to Eurasia and North America (Inda *et al*., 2008, 2014), implying that the ancestral diploid contributing to South American polyploids must have colonized the continent prior to, or concurrent with, polyploidization events. The discovery of this pivotal genome shared across widely separated continental lineages has broad implications not only for understanding *Festuca* evolution but also for practical grass breeding. Since *F. glaucescens* harbours the diploid-like meiotic pairing regulator that governs chromosome pairing in polyploids including *F. arundinacea* and *F. glaucescens* (Jauhar, 1975; Kopecký *et al*., 2009), identifying the equivalent genomic region in South American taxa may provide novel insights into the genetic architecture and evolution of this meiotic control system. The concept of a pivotal genome was first described in Triticeae (*Triticum* and *Aegilops*) by Zohary and Feldman (1962). They speculated that this pivotal genome shared by various allotetraploids of the *Aegilops–Triticum* group remained unaltered by hybridization and served as a buffer facilitating interamphidiploid gene-flow.

The phylogenetic proximity of South American and Eurasian fescues revealed here is consistent with the long-recognized role of South America as a secondary centre of *Festuca* diversification (Dubcovsky and Martinez, 1992; Stančík, 2003; Fernández Pepi *et al*., 2012; Ospina and Picca, 2019). The genus is estimated to have originated approximately 20 million years ago in the Mediterranean–SW Asian region (Darbyshire *et al*., 2003), subsequently colonizing the New World (Inda *et al*., 2008). Our results support this dispersal hypothesis by demonstrating that genomes of South American fescues from subg. *Subulatae* and subg. *Mallopetalon* can be traced to a Eurasian diploid lineage closely related to one of the diploid genomes of the polyploid fescues from subg. *Schedonorus*. This is direct cytogenomic evidence for a shared ancestral genome between South American and Eurasian polyploid fescues, moving beyond earlier morphological and molecular sequence-based inferences of the phylogenetic affinity (Inda *et al*., 2008; Fernández Pepi *et al*., 2012). The pivotal genome detected here illustrates the limitations of standard phylogenetic analyses. Although they can reveal overall relationships among species, most available datasets rely on plastid genomes and multicopy ribosomal markers, which generally represent a single parental lineage or a homogenized nuclear signal (Catalán *et al*., 2004; Inda *et al*., 2008; Fernández Pepi *et al*., 2012). Consequently, they cannot readily distinguish the different diploid parental genomes coexisting within an allopolyploid nucleus. In contrast, comparative GISH enables direct visualization of genome-wide homology across complete chromosome complements, revealing genomic affinities that remain largely hidden in conventional phylogenetic analyses. However, only future whole-genome sequencing and genome assembly of these species will enable the precise characterization of this pivotal genome and its tracking within the evolutionary history of the Loliinae.

The trans-continental genomic affinity described here adds to a growing body of evidence that polyploid fescues from outside the Eurasian core range of subg. *Schedonorus* share phylogenetic histories with Eurasian lineages. Namaganda and Lye (2008) demonstrated, on morphological grounds, that the central African species *F. simensis* Hochst. ex A.Rich. is closely related to polyploid species of subg. *Schedonorus* and proposed its placement within that subgenus, noting its particular morphological proximity to the European *F. gigantea* (L.) Vill., supported by the molecular phylogeny (Namaganda *et al*., 2006). The results presented here are strikingly analogous: just as *F. simensis* points to the dispersal of *Schedonorus*-affiliated lineages into tropical Africa, our data indicate that closely related ancestral genome(s) also reached South America. Whether the African and South American colonization events were independent or represent a single dispersal that subsequently diversified across both regions awaits molecular dating and broader taxon sampling. Nonetheless, the convergence of evidence across different continents underscores that the biogeographical history of polyploid broad-leaved fescues is considerably more complex than previously appreciated.

The discovery of a shared pivotal diploid genome between South American and Eurasian polyploid fescues has direct implications for the study of the diploid-like meiotic pairing regulator in *Festuca*. Unlike the *Ph1* system of wheat or the *PrBn* locus of *Brassica napus* L., both of which function in single doses (Jauhar, 1993; Jenczewski *et al*., 2003; Liu *et al*., 2006), the meiotic chromosome pairing regulator in polyploid fescues is haplo-insufficient (hemizygous-ineffective), a characteristic that sets it apart mechanistically from well-characterized homoeologous pairing suppression systems in other allopolyploids (Jauhar, 1993). This regulator was inherited in *F. arundinacea* from its tetraploid progenitor *F. glaucescens*, and its precise chromosomal location within the two indistinguishable diploid genomes of *F. glaucescens* remains unknown (Kopecký *et al*., 2009). Since the genome of *F. ulochaeta* is closely related to one of these diploid genomes, *F. ulochaeta* and *F. fimbriata* may offer complementary material for fine-mapping and characterizing the pairing regulator. In interspecific hybrids between fescue and ryegrass (*Festuca × Lolium*), genomic instability arising from homoeologous chromosome pairing and recombination is a major obstacle for grass breeding (Kopecký *et al*., 2008; Kopecký and Studer, 2014). A deeper understanding of the meiotic pairing system, informed by the broader evolutionary context provided by this study, could contribute to strategies that better harness the genetic diversity of South American fescues in breeding programmes targeting forage quality, stress tolerance and persistence.

In conclusion, this study, by using comparative GISH, demonstrates clearly that the South American hexaploid fescues *F. ulochaeta* and *F. fimbriata* are genomically closely related to each other and their genome is phylogenetically close to a pivotal diploid-derived genome of Eurasian broad-leaved polyploid fescues from subg. *Schedonorus*. This represents the first cytogenomic evidence for a trans-continental genomic connection between South American and Eurasian polyploid fescues, with important ramifications for our understanding of *Festuca* biogeography, polyploid evolution and the origin of meiotic control systems. Future work involving whole-genome sequencing of South American *Festuca* species, molecular dating and expanded GISH with additional diploid probes will be essential to identify the true ancestral diploid progenitor of this pivotal genome and to fully reconstruct the allopolyploid histories of South American fescues. This will also provide an opportunity for precise dating of the trans-continental migration(s). Another future research direction is the involvement of fine-leaved fescues (subgenus *Festuca*). This subgenus represents the most species-rich group within the genus and diverged from broad-leaved fescues (incl. *Schedonorus*, *Mallopetalon* and *Subulatae* involved in this study) in the Miocene (about 13 Mya; Inda *et al*., 2008, 2014). If our pivotal genome is present in fine-leaved fescues, it might represent an ancient ancestral genome whose origin pre-dates the diversification of the genus *Festuca*. This opens a way for future studies on broad phylogenetic and evolutionary analyses in this largest genus within Poaceae and indicates the potential for intercrossing of distinct European and South American lineages via this shared pivotal genome in breeding programmes.

## Supplementary Material

mcag261_Supplementary_Data

## Data Availability

The data supporting the findings of this study are available in the main manuscript.

## SUPPLEMENTARY DATA

Supplementary data are available at *Annals of Botany* online and consist of the following. Table S1: Origin, chromosome number, ploidy level and genome size of the analysed species (S1A); chromosome length measurements (S1B); and size estimation of pivotal genome in three fescue species (S1C).

## AI ASSISTANCE

DeepL was used for English grammar corrections.
